# Xylariales: First results of mycological exploration in the Sangay and Llanganates National Parks, Ecuador

**DOI:** 10.12688/f1000research.13623.2

**Published:** 2018-05-17

**Authors:** María-Fernanda Guevara, Bence Mátyás, María-Eugenia Ordoñez

**Affiliations:** 1 Biotechnology of Natural Resources, Universidad Politécnica Salesiana, Quito, 170525, Ecuador; 2 Environmental Research Group, Secondary Metabolites and Animal Biotechnology NUNKUY-WAKAN, Universidad Politécnica Salesiana, Quito, 170525, Ecuador; 3 Grupo de Investigación Mentoria y Gestión del Cambio, Universidad Politécnica Salesiana, Cuenca, 010102, Ecuador; 4 Grupo de Investigación Ambiental para el Desarrollo Sustentable- GIADES, Universidad Politécnica Salesiana, Quito, Ecuador; 5 School of Biological Sciences, Pontifical Catholic University of Ecuador, Quito, 170143, Ecuador

**Keywords:** Diversity, ITS, Llanganates, National Park, Sangay, Xylarial

## Abstract

Fungal samples were collected in the Sangay (SP) and Llanganates (LP) National Parks in Ecuador. Sequences of the internal transcribed spacer regions (ITS1-5.8S-ITS2) of the ribosomal DNA of the samples were analyzed.Taxonomic identification of fungi of the order Xylariales was done through phylogenetic analysis using a Maximun Likelihood method. All analyzed collections presented here belong to the genus Xylaria, of these eight belong to PL and two to SP. Four samples were not identified at the species level, suggesting it could be a new species. This data contributes with base information on the biodiversity of the Parks, necessary to design and implement measures for the conservation of fungi in Ecuador.

## Introduction

Sangay (SP) and Llanganates (LP) National Parks in Ecuador are considered as high priority conservation units in the Tropical Andes, due to their high biodiversity and high endemism ^1, 2^. However, their mycological diversity is still unknown. This study aims to contribute to the conservation of fungi, showing the results of their diversity, based on molecular taxonomy, by analyzing the ITS (internal transcribed spacer) regions. ITS is the accepted as primary fungal barcode marker for fungi ^3, 4^. For this, the DNA sequence of specimens of exploratory fungal collections were analyzed within the aforementioned parks. Here we present results exclusively for the Xylariales order, other fungal orders were also collected but are not shown here.

## Methods

### Sequencing and molecular identification

Sample collection was carried out during the months of January and February 2015. The fruiting bodies collected were deposited in the QCAM Fungarium (Catholic University Mycology Collection, Quito). Table 1 displays the collection codes, as stored at the QCAM. The ITS1-5.8SITS2 region was amplified by PCR with primers ITS1F (5’-CTTGGTCATTTAGAGGAAGTAA-3’) ^5^ and ITS4 (5’-TCCTCCGCTTATTGATATGC-3’) ^6^. The amplified fragments were sent for sequencing to Macrogen Inc. (Seoul, South Korea). The forward and reverse sequences obtained for each isolate were assembled in Geneious R8 (Biomatter Ltd. 2005–2012), and submitted to GenBank. Sequence data were analyzed by comparison with sequences available in GenBank. An assignment to the lower taxonomic level was made by direct homology of the consensus sequences with the search results in BLASTn (NCBI) optimized for highly similar sequences (megablast). Alignments that presented 100% coverage and at least 99% identity with a sequence previously reported in GenBank were considered. The results were compared with the previously made morphological identification at the QCAM Fungarium, to check the taxonomic designation.

**Table 1.** Fungi of the order Xylariales collected in the Sangay and Llanganates National Parks in Ecuador. *SNPs: Single nucleotide polymorphisms found between the sample analyzed and the closest hit on the BLASTn search.

QCAM Fungarium Code	GenBank Accession Number	Identification	Sampling location	
			National Park	Altitude (m)
QCAM4663	MG768840	Xylaria enterogena	Llangantes	1370
QCAM4551	MG768839	Xylaria enterogena	Llangantes	1387
QCAM4537	MG768834	Xylaria fissilis	Llangantes	1377
QCAM4540	MG768836	Xylaria schweinitzii	Llangantes	1377
QCAM4232A	MG768832Â	Xylaria telfairii	Sangay	2885
QCAM4550	MG768838	Xylaria sp. 1	Llangantes	1387
QCAM4666	MG768841	Xylaria sp. 1	Llangantes	1379
QCAM4306A	MG768833	Xylaria sp. 2	Sangay	2885
QCAM4545	MG768837	Xylaria sp. 3	Llangantes	1373
QCAM4539	MG768835	Xylaria sp. 4	Llangantes	1377

### Phylogenetic analysis

Sequence data were aligned with Geneious R8 and later manually adjusted with Mesquite version 3.04 ^7^. Public sequences available in GenBank that corresponded to specimens that gave the greatest homology in BLASTn with the sequences of the collected specimens were included. Phylogenetic trees were constructed in Geneious R8 using the PhyML ^8^ plugin for Maximum Likelihood (ML) with a custom substitution model (010230), determined by jModelTest 2.1.4. ^9, 10^, according to Corrected Akaike Information Criterion (AICc) ^11, 12^. A bootstrap of 1000 replica was used.

## Results

**Figure 1.**
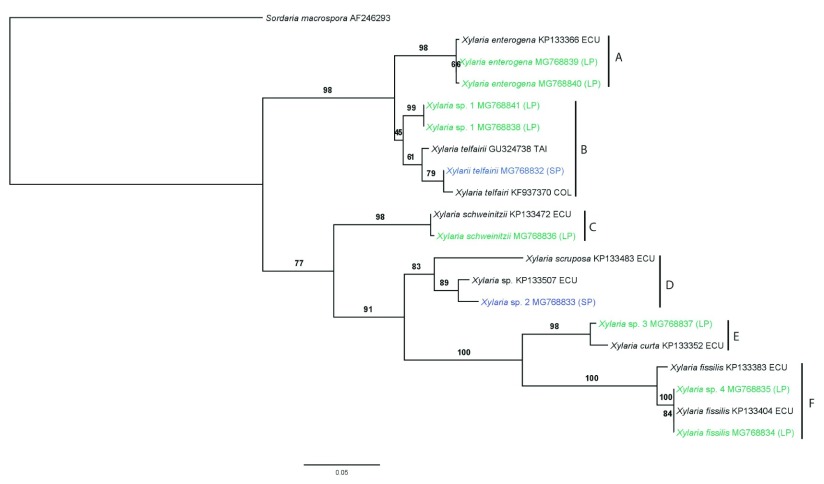
Maximum likelihood phylogenetic tree of specimens of the Xylariales order obtained in Sangay and Llangantes National Parks, based on sequences of the ITS (internal transcribed spacer) region.

All the specimens analyzed were of the genus Xylaria. The eight specimens from Llanganates National Park were identified as X. enterogena, X. fissilis, X. schweinitzii, X. telfairii and three unidentified species. For the two samples from Sangay National Park, one was X. telfairi and the other was an unidentified species. Differences in the number of samples found at each park could be due to the sampling effort, and not necessarily to the richness of the Xylareales in the Parks. The unidentified species were different in each park. The analysis shows that there are no shared species of Xylaria at the two sampled sites ( Table 1), this is important for conservation decision making. The phylogenetic relationships recovered from the analysis of the ITS sequences ( Figure 1) shows two major groups. The first major group, composed by clades A and B, is well supported, it includes specimens from LP and PS. Clade A includes all X. entogena specimens and Clade B includes all X. telfairii specimens, and Xylarya sp.1 specimens. It is possible that Xylarya sp.1 might belong to the X. telfairii group, but due to the differences among the sequences it is likely a different species. In the second major group, clade C is sister to clades D, E and F. This major group includes specimens from both LP and SP. Clade C includes all X. schweinitzii specimens. Clade D includes Xylaria sp. 2. The closest sequence to Xylaria sp. 2 from SP was a previously reported collection also from Ecuador ^13^ in a cloud forest in the province of Imbabura, that was also identified only at the genus level. Clade E shows Xylaria sp. 3, the closest sequence to this individual belongs to the same previously reported study ^13^, identified only at the genus level. Clade F includes Xylaria fissilis sequences from LP and one from 13. Clade F also includes Xylaria sp. 4, an unidentified specimen. The number of nucleotide differences (SNPs) between the sample and the closest hit on the BLASTn search suggests that these specimens may belong to new species ( Table 1). Additional loci and more detailed morphological analyses are needed to determine this. The genus Xylaria is probably the largest in the family Xylariaceae, with 35 estimated genera ^14^, but the real number remains unknown ^15^. Studies related to the biological diversity of this order in the National Parks of Ecuador are scarce, more systematic field studies would surely reveal a greater diversity of families, genera and species within the Xylariales in SP and LP, as well as other regions and protected areas of Ecuador, especially if we take into account the cosmopolitan distribution of Xylaria ^13^. In fact, new fungal species in SP, belonging to the Agaricales, have recently been described ^16^.

These entire unidentified specimens might represent new species. Additional loci and more detailed morphological analyses are needed to determine this. The genus Xylaria is probably the largest in the family Xylariaceae, with 35 estimated genera ^13^, but the real number remains unknown ^15^. Studies in relation to the biological diversity of this order in the National Parks of Ecuador are scarce, more systematic field studies would surely reveal a greater diversity of families, genera and species within the Xylariales in SP and LP, as well as other regions and protected areas of Ecuador, especially if we take into account the cosmopolitan distribution of Xylaria ^13^. In fact, new fungal species in PS, belonging to the Agaricales, have recently been described ^16^.

## Conclusions

The results obtained allow us to establish a baseline for the biological diversity of the Xylariales in SP and LP, an important step to the conservation of fungi. This is the main contribution of this study. We found four species of Xylaria: X. enterogena, X. telfairii, X. schweinitzii, and X. fissilis, and four potential new species based on ITS sequences divergence; the species found in LP are different from those found in SP. However, there is much more to discover. A huge and complex task is pending. To advance our understanding of the Kingdom Fungi we must start by deciphering the diversity of species present in these sites.

## Data availability

The sequencing data are available on the NCBI Genbank webpage: Xylaria enterogena:

https://www.ncbi.nlm.nih.gov/nuccore/MG768840

https://www.ncbi.nlm.nih.gov/nuccore/MG768839

Xylaria fissilis: https://www.ncbi.nlm.nih.gov/nuccore/MG768834

Xylaria schweinitzii: https://www.ncbi.nlm.nih.gov/nuccore/MG768836

Xylaria telfairii: https://www.ncbi.nlm.nih.gov/nuccore/MG768832

Xylaria sp. 1: https://www.ncbi.nlm.nih.gov/nuccore/MG768838

https://www.ncbi.nlm.nih.gov/nuccore/MG768841

Xylaria sp. 2: https://www.ncbi.nlm.nih.gov/nuccore/MG768833

Xylaria sp. 3: https://www.ncbi.nlm.nih.gov/nuccore/MG768837

Xylaria sp. 4: https://www.ncbi.nlm.nih.gov/nuccore/MG768835
