# Supplementary material for: Xylariales: First results of mycological exploration in the Sangay and Llanganates National Parks, Ecuador
Source: F1000Res. 2018 May 17;7:222. Originally published 2018 Feb 23. [Version 2] doi: 10.12688/f1000research.13623.2 (PMC6051188; doi:10.12688/f1000research.13623.2)
Supplement: Peer review history

# Peer review: Referee response for version 2

**DOI:** 10.5256/f1000research.16220.r35790

**Published:** 2018-07-16

**Authors:** Juan Enrique Tacoronte Morales

**Affiliations:** 1 Central University of Ecuador, Quito, Ecuador

I have read this submission proposal and all related information reported in (introduction, methodology, results, discussion and cited references). Considering my expertise in secondary metabolites and bioactive natural products isolated from invertebrates (defensive secretions and applied chemical ecology) and fungi (volatile organic compounds) I believe that I could analyze the reported information data with an appropriate, conceptual and methodological level to confirm that this report devoted to mycological exploration of Ascomycota-Xylariales in tropical Andean region has a quite recognized scientific standards for being published in *F1000Research.*

 In this context, please, I have some considerations regarding the submitted article:

 - it could be of great significant and relevance to evaluate some biogeochemical parameters of eco-geographical collecting zones for potential determining the zonal distribution of Xylariales in these areas, including any potential correlation with tree diversity.

**-**It would have been highly didactic, and recommended, the inclusion, in the proposal submitted, of some photographs in real time, taken during the collection phase in the national parks, and some, comparative ones, taken during the preparation phase in the lab (for morphological purposes only!)

I have read this submission. I believe that I have an appropriate level of expertise to confirm that it is of an acceptable scientific standard.

# Peer review: Referee response for version 2

**DOI:** 10.5256/f1000research.16220.r34114

**Published:** 2018-05-29

**Authors:** Francisco J. Flores

**Affiliations:** 1 Departamento de Ciencias de la vida y la Agricultura, Universidad de las Fuerzas Armadas–ESPE, Sangolquí, Ecuador

There are still several issues that need to be addressed before indexing. Please see the file in the provided link for specifics:

Click here for annotated PDF

I have read this submission. I believe that I have an appropriate level of expertise to confirm that it is of an acceptable scientific standard, however I have significant reservations, as outlined above.

# Peer review: Referee response for version 2

**DOI:** 10.5256/f1000research.16220.r34113

**Published:** 2018-05-24

**Authors:** D. Jean Lodge

**Affiliations:** 1 Department of Agriculture, USDA Forest Service, Luquillo, PR, USA; 2 Department of Plant Biology, University of Georgia, Athens, GA, USA

Abstract:

 4th line from top - change 'done' to 'inferred' because you can't be sure of an identification based on ITS blast.

 3rd line from bottom, change sentence beginning "This data" to These data provide baseline information"

 The Xylaria sp. 3 is still listed as unidentified. The molecular phylogeny indicates this is likely X. curta. X. curta is expected from that area and has been collected before in that part of Ecuador. It is easily identified in photographs, with confirmation by examining spores and ascus plugs under a microscope. It is not clear why this point was not addressed in revisions, though it would require renumbering of the unidentified species in tables and figures and text. Also, Xylaria sp. 4 appears to be X. fossils based on the phylogeny.

 The abstract says there were 4 unidentified Xylaria species, but if sp. 3 is X. curta and sp. 4 is X. fossils, there are only two suspected undescribed species - sp. 1 and sp. 2.

 The conclusions say there were four unnamed species also, but X. fissilis is among the named species based on one collection, but sp. 4 has an identical sequence to the named one, so that is also X. fissilis and not a new species.

 The manuscript should not be indexed until the authors reduce the number of undescribed species from 4 to 2 and make the corresponding changes to the text throughout.

I have read this submission. I believe that I have an appropriate level of expertise to confirm that it is of an acceptable scientific standard, however I have significant reservations, as outlined above.

# Peer review: Referee response for version 1

**DOI:** 10.5256/f1000research.14800.r32603

**Published:** 2018-04-19

**Authors:** D. Jean Lodge

**Affiliations:** 1 Department of Agriculture, USDA Forest Service, Luquillo, PR, USA; 2 Department of Plant Biology, University of Georgia, Athens, GA, USA

Introduction. Because the aim is to compare these two parks in Ecuador, it is important that the elevation ranges of each are included, as well as the distance between the two parks.

 Methods, p. 3, last line seems to be an incomplete sentence. It ends with ‘BLASTn’ (NCBI) optimized for highly similar sequences (megablast), alignments that presented 100’

 It is probably meant to say ‘overlapped 100%.’

 Results: Text for Clade E does not match the phylogram. The text says that sp. 3 was related to another collection identified only at the genus level. However, the phylogram shows a well-supported clade (98% BS) comprised of sp. 3 and *X. curta* KP133352 ECU. I would expect *X. curta* to be reasonably common there. There is either an error in identification of KP133352 ECU or the text in resutls needs to be changed, and the abstract and first line of results and conclusions also need to be changed to include *X. curta*.

 That also means that the unidentified species need to be renumbered in the text, table, and phylogram, with sp. 4 becoming sp. 3.

 There are likely 3 undescribed species included in this study. The journal allows for photographs, and photos should be added to the manuscript. Even if photos were not taken of the fresh specimens, photos of the dried specimens can be used, and are very helpful. If photos of the asci, especially the ascus plug stained with iodine/Melzer’s reagent, and photos of the ascospores showing the germ slits, that would be very helpful also for future work. The ascospore length and width ranges, the shape and extent of the germ slit, and the shape and size of the ascus plug would be helpful additions.

I have read this submission. I believe that I have an appropriate level of expertise to confirm that it is of an acceptable scientific standard, however I have significant reservations, as outlined above.

# Peer review: Referee response for version 1

**DOI:** 10.5256/f1000research.14800.r31232

**Published:** 2018-03-01

**Authors:** Francisco J. Flores

**Affiliations:** 1 Departamento de Ciencias de la vida y la Agricultura, Universidad de las Fuerzas Armadas–ESPE, Sangolquí, Ecuador

The article "Xylariales: First results of mycological exploration in the Sangay and Llanganates National Park, Ecuador " provides internal transcribed spacer sequences from *Xylaria *isolates **collected from Ecuadorian national parks. Information about fungal diversity in Ecuador is still scarce, which makes this manuscript relevant. Nevertheless, I have several concerns, mainly:

- Phylogenetic analysis using only the ITS sequence is very superficial, and, most likely, it won´t show an accurate representation of the real evolutionary relationships between isolates.
- There is no mention about the morphological characteristics of isolates. Is the morphology congruent with the genotype?

The manuscript needs major revision to improve clarity.

 Several suggestions are made in the file linked below.

https://f1000researchdata.s3.amazonaws.com/linked/196501.Bence_Matyas_rev.pdf

I have read this submission. I believe that I have an appropriate level of expertise to confirm that it is of an acceptable scientific standard, however I have significant reservations, as outlined above.
